# Redox imbalance induced by docetaxel in the neuroblastoma SH-SY5Y cells: a study of docetaxel-induced neuronal damage

**DOI:** 10.1080/13510002.2021.1884802

**Published:** 2021-02-09

**Authors:** Lucia Micheli, Giulia Collodel, Elena Moretti, Daria Noto, Andrea Menchiari, Daniela Cerretani, Sergio Crispino, Cinzia Signorini

**Affiliations:** a Department of Medical and Surgical Sciences and Neurosciences, University of Siena, Siena, Italy; b Department of Molecular and Developmental Medicine, University of Siena, Siena, Italy; c Department of Business and Law, University of Siena, Siena, Italy; d Scientific Direction ASSO, Siena, Italy

**Keywords:** Apoptosis, cellular antioxidants, chemobrain, docetaxel, isoprostanes, SH-SY5Y cells, tubulin, transmission electron microscopy

## Abstract

**Objectives:**

In cancer survivors, chemotherapy-associated adverse neurological effects are described as side effects in non-targeted tissue. We investigated the role of redox-imbalance in neuronal damage by a relative low dose of Docetaxel (DTX).

**Methods:**

The neuroblastoma cells (SH-SY5Y cells) were exposed to DTX at a dose of 1.25 nM for 6 h. Antioxidant defenses (i.e. ascorbic acid, glutathione, and catalase) and lipid oxidation products (i.e. F_2_-isoprostanes) were evaluated. To investigate cell ultrastructure and tubulin localisation, transmission electron microscopy (TEM) and immunofluorescence techniques were applied.

**Results:**

In the SH-SY5Y cells, DTX induced a significant reduction of total glutathione (*P* < 0.001) and ascorbic acid (*P* < 0.05), and an increase in both total F_2_-Isoprostanes (*P* < 0.05) and catalase activity (*P* < 0.05), as compared to untreated cells. Additionally, TEM showed a significant increase in cells with apoptotic characteristics. Immunolocalisation of tubulin showed a compromised cytoskeletal organisation.

**Discussion:**

The investigated sublethal dose of DTX, to which non-targeted cells may be exposed throughout the duration of chemotherapy treatment, induces a redox imbalance resulting in a specific modulation of the antioxidant response. This study provides new insights into DTX-induced cellular mechanisms useful for evaluating whether the concomitant use of antioxidants associated with chemotherapy mitigates chemotherapy side effects in cancer survivors.

## 1. Introduction

An oxidant/antioxidant imbalance is implicated in tumour progression [1] and is also relevant to the side effects of chemotherapy drugs [2]. It is also believed that the formation of free radicals is involved in the mechanisms of chemotherapy drugs against cancer cells [3]. Therefore, researchers discuss whether patients may benefit, in alleviating side effects from toxic chemotherapies, from an antioxidant supplementation while undergoing chemotherapy [3]. As long-term side effects of chemotherapy, an array of cognitive impairments and alterations in brain structure and function, such as changes in the hippocampus, neurogenesis [4], white matter [5,6], and cerebral blood supply [7], have been described. Such chemotherapy-induced cognitive impairment (CICI) [8], also called chemobrain or chemofog [9,10], continues for up to 10 years after chemotherapy treatment [11]. Cognitive deficit is a condition that strongly affects the follow-up of cancer survivors. Nevertheless, there are no effective interventions to prevent chemobrain. Although cytokine modulation has been reported to play a key role in the pathophysiology of chemobrain [12], CICI has also been related both to increased production of free radicals [13] and to a greater susceptibility to oxidative stress [14,15]. For a clear demonstration of the relationship between chemotherapy and cognitive impairment, reliable biomarkers seem to be lacking [16]. Nevertheless, since the 1990s the connection between oxidative stress and cognitive impairment has been highlighted [17], and growing evidence to support a link between high ROS levels and both alteration of antioxidant system and ROS-induced cell injury has become available [15] and been reviewed [17].

Currently, docetaxel (DTX) is an antineoplastic agent widely used, in mono- or combination drug therapies, to treat several solid tumours [18]. Like other taxanes, DTX is a potent anti-mitotic agent that acts by binding to tubulins/microtubules that have a key role in cell division. Consequently, the suppression of microtubule dynamics results in the blockade of cell mitosis, leading to apoptosis [19]. It is known that DTX side effects on the brain, as non-targeted tissue, have been related to oxidative stress [20]. In this connection the protective role of melatonin and selenium in mitochondrial oxidative stress has been reported in DTX-treated mice [21].

Some mechanisms have been hypothesised for the relationship between oxidative stress and CICI. In rats, a long-term cognitive dysfunction was observed following docetaxel treatment [22], and the efficacy of resveratrol in preventing chemobrain by a combination of docetaxel, adriamycin, and cyclophosphamide was demonstrated in mice [23].

Our purpose was to investigate the role of the antioxidant/oxidant parameters in DTX-induced neuron damage, as a relevant factor in the managing and prevention of CICI. To this end, we analysed several biochemical parameters (total glutathione, ascorbic acid, catalase activity, and F_2_-IsoPs), and microscopic criteria (ultrastructure and cytoskeletal organisation) in neuroblastoma SH-SY5Y cells treated with a sublethal dose of DTX. Thus, the SH-SY5Y cells, currently considered a well-established neuronal cell model and used to explore nervous system impairment [24,25], were exposed to a DTX dose to mimic the status of non-target cells exposed to side effects of chemotherapy.

## 2. Materials and methods

### 2.1. Solvents and Reagents

All solvents used were of HPLC-MS grade and purchased from Sigma-Aldrich (Dorset, UK). Ultrapure water (18.2 MΩ was obtained from a Milli-Q integral water purification system (EMD Millipore, MA, USA). Standards for antioxidant assays were purchased from Sigma-Aldrich (Dorset, UK). For cell culture, RPMI-1640 medium, phosphate buffered saline (PBS), L-glutamine, Penicillin–Streptomycin, HEPES, and FBS were purchased from EuroClone SPA (Italy).

### 2.2. Neuroblastoma SH-SY5Y cell culture

For this study, the human neuroblastoma SH-SY5Y cell line was grown in 25 cm^2^ flasks containing 5 ml Roswell Park Memorial Institute medium (RPMI 1640), containing 10% (vol/vol) heat-inactivated fetal bovine serum (FBS), 100 U/ml penicillin, and 100 μg/ml streptomycin, 2.05 mM L -glutamine, and 25 mM 4-(2-hydroxyethyl)-1-piperazineethanesulfonic acid (HEPES). Cell cultures were kept in a humidified incubator (Galaxy B, RS Biotech) at 37°C with 5% CO_2_, and when the cultures reached confluence (70–80% of the surface of the flask covered by cell monolayer), the medium was removed, the cells were washed twice with phosphate-buffered saline (PBS) and split with 2 ml of trypsin (0.25%) / 0.53 mM ethylenediaminetetraacetic acid (EDTA) solution, at 37°C for 2 min. The cells were recovered in 10 ml of complete medium, transferred in a sterile tube, and centrifuged at a low speed (2,000 *g* for 7 min at room temperature). Afterward, the supernatant was removed and the cell count (in triplicate) was performed [26].

The pellet, properly disrupted, was suspended in complete culture medium and transferred to a 75 cm^2^ flask to proceed to the next steps.

All technical procedures described were carried out under a vertical laminar flow cabinet Steril VBH biohazard cabinet class II (Steril, Italy).

### 2.3. Cell viability assay. 3-(4,5-dimethylthiazol-2-yl)-2,5-diphenyltetrazolium bromide (MTT) test

To establish experimental conditions for exposure time and DTX concentration, the cells were seeded in triplicate in 96-well plates at a concentration of 10,000 cells/well and grown for 24 h in 200 µl of the RPMI complete medium.

After 24 h, 100 µl of the medium was removed and the cells were treated with 100 µl of medium containing a concentration of DTX ranging from 0.1 nM to 10 µM (DTX group), or with 100 µl of medium containing ethanol at a concentration ranging from 10 nM to 1 mM (vehicle group), or with 100 µl of medium only (control group) for 3, 6, 12, 24, 48, and 72 h (triplicate plates per experiment).

After incubation with different concentrations of DTX, at different times (reported above), cell viability was detected using 3-(4,5-dimethylthiazol-2-yl)-2,5-diphenyltetrazolium bromide (MTT) assay.

The purple formazan crystals were dissolved in 0.04 N HCl in isopropanol and the absorbance was measured on a microplate reader (Bio-Rad iMark Reader, Italy) at a wavelength of 570 nm.

### 2.4. Determination carried out in cells

#### 2.4.1. Treatment of the SH-SY5Y cell culture- incubation in the presence of DTX or vehicle drug

To induce a neuronal damage by DTX, in absence of a high grade of cell death, a low dose of the drug (1.25 nM) with an exposition of 6 h was the experimental protocol defined on the basis of the cell viability assay above described.

For this study, the SH-SY5Y cell line was grown in 75 cm^2^ flasks with RPMI medium complete in a humidified incubator at 37°C with 5% CO_2_. When the cultures reached confluence (cells were approximately 70–80% confluent), the medium was removed and the cells were split and seeded into new flasks at 5 × 10^5^/ml. The cells were plated in three plates for each group.

After 24 h, the culture medium was removed and cells were treated with a medium containing DTX at a concentration of 1.25 nM (DTX group) or with a medium containing ethanol at a concentration of 10 nM (vehicle group), or else with medium only (control group), and incubated for 6 h. At the end of the incubations, the medium was removed and cells were washed twice with PBS. Then the cells were recovered by gentle scraping, and centrifuged at 1,000 rpm for 5 min at 4°C. The pellets were suspended in PBS and the cells were counted to determine the total number of cells in suspension. Afterward, the cells, immersed in an ice bath, were lysed by sonication for 10 s (Vibracell Sonicator; amplitude 60, 25 W), and immediately frozen at −80°C until they were used for the analyses described below.

For ultrastructural investigations, the cells were seeded into 6-well plates at a density of 2 × 10^5^ cells/well and then treated with DTX (1.25 nM) or vehicle (medium containing ethanol at a concentration of 10 nM), or with medium only, for 6 h. After incubation, the medium was removed, the cells were washed with PBS three times and then prepared to be used in different analyses as described below.

#### 2.4.2. Detection of oxidised and reduced glutathione

An aliquot of the pellet lysed was thawed by adding an equal volume of 10% metaphosphoric acid and centrifuged at 2,000 *g* for 10 min at 0°C. In the supernatant, total glutathione, reduced glutathione (GSH) plus oxidised glutathione (GSSG), was quantified using a micro-assay procedure [27] based on the catalytic action of GSH or GSSG in the reduction of Ellman reagent (5,5′-dithiobis-[2-nitrobenzoic acid], DTNB) by a mixture of triphosphopyridine nucleotide (TPNH) and yeast glutathione reductase. The absorbance was detected at 415 nm, for 2 min on a microplate reader (Bio-Rad’s iMark Microplate Absorbance Reader). Results were expressed as ng/10^6^ cells. The 2-Vinylpyridine, as an alkylating agent that masks GSH reduced form leaving only GSSG, was used for the oxidised glutathione determination. This substance does not inhibit the enzyme glutathione reductase and the colorimetric assay of glutathione oxidised was performed as for total GSH.

#### 2.4.3. Detection of catalase activity

Immediately after thawing, an aliquot of cell lysate was added to an equal volume of ice-cold phosphate buffer (0.125 M, pH 7.4) containing 1 mM EDTA and then centrifuged at 4,000 *g* for 15 min at 4°C. Catalase activity was determined with a micro-assay procedure previously described by Johansson and Borg [28]. One unit of catalase activity is defined as the amount of enzyme that will cause the formation of 1  nmol of formaldehyde per minute at 25°C. Results were expressed as U/10^6^ cells.

#### 2.4.4. Ascorbic acid assay

An aliquot of the lysed pellet was thawed by adding an equal volume of 10% metaphosphoric acid and centrifuged at 2,000 g for 10 min at 0°C. Ascorbic acid (AA) levels were measured by HPLC method as described by Ross [29] with minor modification. AA was quantified by UV reverse-phase HPLC using a Waters 600 E System Controller HPLC (Milford, MA, USA) equipped with a dual λ absorbance UV-visible detector with the wavelength set at 262 nm (Waters 2487 Milford, MA, USA). Chromatographic separations were accomplished on an ultrasphere ODS column (250 mm × 4.6 mm i.d., 5 µm particles diameter), reversed phase (Beckman, San Ramon, CA, USA). The mobile phase was composed of a mixture of acetonitrile: water (49:51, v/v) at a flow rate of 0.8 ml/min. Agilent 3395 integrator (Agilent Technologies, USA) was used to process chromatography data and the ascorbic acid concentrations (nmol/ml) were calculated by peak areas.

#### 2.4.5. F_2_-isoprostane determination

Isoprostanes, which are used as biomarkers of oxidative damage, are a large family of compounds derived from non-enzymatic oxidation of polyunsaturated fatty acids (PUFAs). Isoprostanes provide unique information about the precursor of the targeted PUFAs. In particular, F_2_-isoprostanes (F_2_-IsoPs), a series of prostaglandin F_2_-like compounds generated by non-enzymatic free radical-initiated peroxidation of esterified arachidonic acid in membranes, are currently detected to evaluate a condition of lipid peroxidation [30]. F_2_-IsoPs, initially formed *in situ* on phospholipids (esterified F_2_-IsoPs), once released into the circulation as unesterified F_2_-IsoPs (free F_2_-IsoPs), are capable of eliciting biological responses in different cell types, and of playing role in normal physiology [30–32].

In the neuroblastoma SH-SY5Y cell line, the quantification of F_2_-IsoPs was performed by gas chromatography/negative ion chemical ionisation tandem mass (GC/NICI–MS/MS) [33].

For the determination of total (sum of free and esterified) F_2_-IsoPs, cell lysates (by sonication, as described above) were frozen in the presence of 100 μM butylhydroxytoluene (BHT). To perform the assays, samples were thawed and basic hydrolysis was carried out in the presence of 1N KOH by means of incubation at 45°C for 45 min. Such incubation was followed by the addition of 1N HCl, and tetradeuterated prostaglandin F_2α_ (PGF_2α_-d_4_) was added (500 pg), as an internal standard. Afterward, ethyl acetate (10 ml) was added to extract total lipids by vortex mixing and centrifugation at 1,000 g for 5 min at room temperature. In the following purification procedures, each total lipid extract was transferred to an NH_2_ cartridge (500 mg Sorbent per Cartridge, 55–105 μm particle size, 6cc, Waters, USA) and procedures of conditioning, washing, and elution were performed. Briefly, the NH_2_ cartridge was conditioned with hexane (5 ml), each eluate was loaded on it, and washes were carried out with 10 ml of hexane: ethyl acetate (30:70, v/v), 10 ml acetonitrile: water (9:1, v/v) and 10 ml acetonitrile. The final elution was a mix of ethyl acetate: methanol: acetic acid (10:85:5, v/v/v, 5 ml), and the collected eluate was first evaporated under nitrogen at 40°C [34, 35].

For free F_2_-IsoP evaluation, a volume of 2 ml acidified water (water acidified at pH 3 by adding HCl 1M to double distilled water) was added to each cell lysate, also containing 100 μM BHT. Each sample was applied on a C_18_ cartridge (500 mg Sorbent per Cartridge, 55–105 µm particle size, 6cc, Waters, USA) previously preconditioned with methanol (5 ml) and water (5 ml), and sequentially washed, after loading the sample, with 10 ml water (water acidified at pH 3 by adding HCl 1M) and 10 ml water: acetonitrile (85:15, v/v). Hexane: ethyl acetate: propan-2-ol (30:65:5 v/v/v, 5 ml) mix was used for the final eluate. The C_18_ eluate was subsequently transferred to an NH_2_ cartridge for a further purification consisting of the conditioning, washing, and elution procedures [33,36].

For free and total F_2_-IsoP assays, all eluates collected from each NH_2_ cartridge were evaporated under nitrogen at 40°C. Subsequently, two derivatisation processes were carried out. First, each sample was incubated, at 40°C for 45 min, in the presence of pentafluorobenzyl bromide (40 μl, 10% in acetonitrile); lastly, incubation at 45°C for 1 h in the presence of 50 μl of N,O-bis (trimethylsilyl)trifluoroacetamide and 5 μl of diisopropylethylamine (10% in acetonitrile) was performed [33]. Subsequently, all F_2_-IsoP determinations were carried out by gas chromatography/ negative ion chemical ionisation tandem mass spectrometry (GC/NICI-MS/MS) analysis. Each derivatised sample was injected (2 μl) into the gas chromatograph (Trace GC and PolarisQ, Thermo/Finnigan, USA) set at splitless mode (2 min), and the oven temperature was increased from 175°C to 270°C (30°C/min). Helium was used as the carrier gas (1 ml/min) and the chromatography was performed using a SPB 1701 GC capillary column (Supelco, 30 m × 0.25 mm i.d., 0.25 μm film thickness). The reagent gas for the chemical ionisation was methane set to 2.0 ml/min flow rate. For F_2_-IsoP GC/NICI–MS/MS analysis, the measured ion was the product ion at *m/z* 299 derived from the [M-181]^−^ precursor ions (*m/z* 569) produced from 15-F_2t_-IsoP, one of the most represented isomers of F_2_-IsoPs generated by free radical-induced arachidonic acid oxidation [33]. The product ion at *m/z* 303, derived from the [M-181]^−^ precursor ion (*m/z* 573), was detected and referred to the internal standard PGF_2α_-d_4_ [33].

The quantitation of free and total F_2_-IsoPs was determined by relating the peak area of 15-F_2t_-IsoP (Cayman Chemical, Item No. 16350) to the deuterated internal standard (Cayman Chemical, Item No. 316010) peak area of the calibration curves constructed. The amounts of F_2_-IsoPs esterified to cellular lipids were estimated as the difference between total and free F_2_-IsoP levels.

#### 2.4.6. Annexin-V and Propidium iodide labelling

All the procedures were carried out as in previous investigations in cultured cells [37]. For assessment of apoptosis and necrosis, treated and control (untreated or vehicle-treated) cells were labelled using the Vybrant Apoptosis Assay kit (InvitrogenLtd, UK) according to the manufacturer’s instructions. At the end of the treatment period, the control (untreated) and treated cells were harvested and washed with cold PBS at 1,200 rpm for 5 min and adjusted to a concentration of 1 × 10^6^ cells/ml with annexin binding buffer (ABB). For each cell suspension, 5 µl of annexin-V conjugated to fluorescein isothiocyanate dye (AnV-FITC) and 1 µl propidium iodide (PI) working solution (100 mg/ml) were added and incubated for 15 min at room temperature. After a careful wash with ABB, a drop of the cell suspension was smeared on each glass slide. Slides were mounted with DABCO (Sigma-Aldrich, Milan, Italy). Observations and photographs were made with a Leitz Aristoplan (Leica, Wetzlar, Germany) light microscope equipped with a fluorescence apparatus. One hundred cells from each sample were examined. This assay is based on staining cells with AnV-FITC (green fluorescence), which make it possible to recognise the translocation of phosphatidylserine (PS) from the inner side of the plasma membrane to the outer layer during the first stage of the apoptotic process, and simultaneously with the non-vital dye PI (red fluorescence), which can penetrate necrotic cells with broken membranes. All experiments were carried out in triplicate. The results are expressed as the percentage of cells that were apoptotic and necrotic.

#### 2.4.7. Immunoﬂuorescence

Immunofluorescence investigations in cultured cells were performed as previously described [38]. The neuroblastoma SH-SY5Y cells were grown on coverslips in medium with phenol red composed of 10% fetal calf serum, 200 U/mL penicillin, 200 U/mL streptomycin, and 2 mM glutamine, at a density of 1 × 10 cell/mL. After treatments, all the samples, treated and controls, were washed in PBS and ﬁxed in methanol /acetone for 20/10 min at −20 °C, respectively.

Subsequently, the samples were saturated for 20 min at room temperature with PBS–Bovine Serum Albumin (BSA, 1%) containing Normal Goat Serum (NGS, 5%) and then incubated overnight at 4°C with anti-β-tubulin mouse monoclonal antibodies (clone TUB 2.1, Sigma–Aldrich), diluted 1:100 in PBS/0.1% BSA/1% NGS. The reaction was revealed by a goat anti-mouse FITC antibody (Santa Cruz Biotechnology), diluted 1:100 in PBS/0.1% BSA/1% NGS, for 1 h at room temperature. Finally, the samples were washed three times in PBS and the coverslips mounted with DABCO (Sigma-Aldrich, Milan, Italy). Incubation with the primary antibody was omitted in control samples. Nuclei were stained with 1 µg/mL DAPI (4′,6-diamidino-2-phenylindole) for 10 min after removal of secondary antibodies. Fluorescence was observed with Leica DMI 6000 (Leica Microsystems, Germany); images were acquired and analysed with Leica AF6500 Integrated System for Imaging and Analysis (Leica Microsystems, Germany). At least 100 cells from each group were evaluated.

#### 2.4.8. Transmission electron microscopy (TEM)

As previously described in studies carried out in cultured cells [37,38], for TEM examination, both the treated and control neuroblastoma SH-SY5Y cells, were ﬁxed in cold Karnovsky ﬁxative and maintained at 4°C for 2 h. Then, cell samples were washed in 0.1 mol/L cacodylate buffer (pH 7.2) for 12 h, post-ﬁxed in 1% buffered osmium tetroxide for 1 h at 4°C, then dehydrated in a graded ethanol series, and embedded in Epon–Araldite. Ultra-thin sections were cut with a Supernova ultramicrotome (Reickert Jung, Vienna, Austria), mounted on copper grids, stained with uranyl acetate and lead citrate, and then observed and photographed with a Philips CM 12 (Philips Scientifics, Eindhoven, The Netherlands), an CE.M.E, CNR (via Madonna del Piano, 10, 50019 Sesto Fiorentino, Italy). At least 100 cells from each group were evaluated.

### 2.5. Statistical analysis

Statistical analysis was performed according to the current statistical methods in medical research [39].

All experiments were independently repeated at least three times to confirm results, using multiple wells replicates or multiple experimental runs. Data represent the mean ± standard deviation of three independent experiments. No statistically significant differences were found between vehicle-treated and untreated cells; therefore, these data were pooled in the control groups.

Statistical analysis was performed using SPSS v.19 (Chicago: SPSS Inc.) Kolmogorov–Smirnov test was used for assessing whether data were normally distributed. Student’s t-test was used when two independent groups were compared. Analysis of data was carried out using one-way ANOVA followed by post hoc tests. The values were considered significantly different when *P*≤0.05.

The correlation between the investigated variables was assessed using Spearman’s rank correlation coefficient (rho).

## 3. Results

### 3.1. Neuroblastoma SH-SY5Y cell toxicity induced by DTX

The cytotoxic effects of DTX were examined in order to select the DTX dose that does not involve lethal effects in more than 50% of the cells.

**Figure 1.**
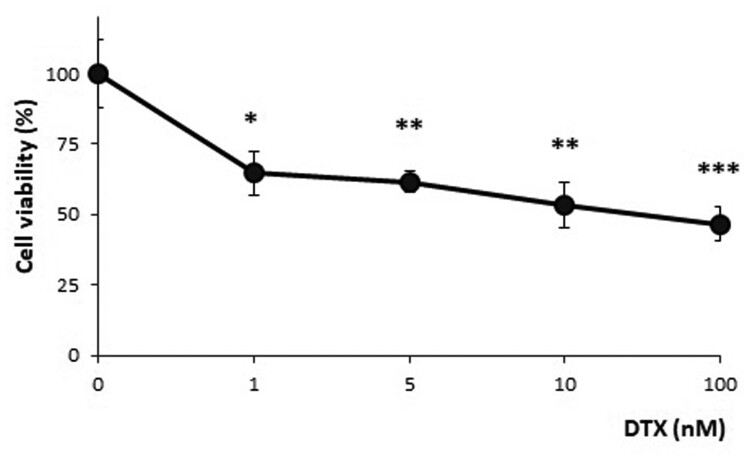
Cell viability after incubation in the presence of different DTX concentrations (1, 5, 10, and 100 nM) for 6 h. MTT assay results show a progressive decline in live cells with increasing concentrations of DTX. The results indicated statistical differences (*P < 0.05; **P < 0.01 and, ***P < 0.001) between DTX-treated (at different doses) and control samples (ANOVA with Tukey’s post hoc test). The results were the means of three independent experiments performed in triplicate.

In preliminary experiments, the SH-SY5Y cell line was treated with DTX at different doses ranging from the lower non-lethal doses (0.1 nM) to the lethal dose (10 µM) for different times of incubation (6, 12, 24, 48, and 72 h), data not shown. Based on these preliminary results, an incubation time of 6 h was selected as the exposure time to DTX to investigate, and a more restricted DTX dose range, from 1 nM to 100 nM, was tested (Figure 1). Although DTX 1 nM was the lowest concentration of DTX tested in MTT, the sublethal dose of DTX was fixed at 1.25 nM. This dose was just slightly higher than the lowest cytotoxic dose, in order to avoid a high degree of cell lethality, while mild oxidative damage and apoptosis were present (described below).

In particular, cell treatment with the selected DTX dose for 6 h results in 65% cell viability (35% mortality), and the cell viability was significantly lower in the DTX treated sample when compared to the control group (vehicle-treated and untreated cells) (*P* < 0.001).

### 3.2. Nuclear and cytoskeletal features

**Figure 2.**
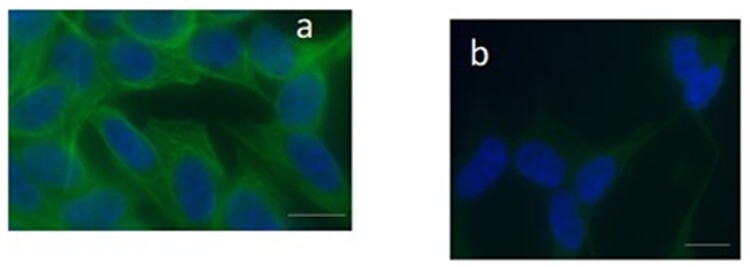
Immunofluorescence staining in the neuroblastoma SH-SY5Y cells using a monoclonal anti-tubulin antibody. In panel a, untreated cells are displayed and the label shows a clear localisation in the cytoskeletal microtubules (green). In panel b, the fluorescent signal appears weak and limited in the reduced cytoplasm, losing its organisation in cells treated with DTX. The nuclei (DAPI blue-fluorescent DNA stain) appeared altered, panel b. Bar 20 μm.

Fluorescent microscopy analysis showed, in the untreated SH-SY5Y cells, that tubulin protein was organised in filaments from the nucleus to the periphery of the cytoplasm, showing in 95.33 ± 1.15% of analysed cells an intense signal (Figure 2(a)). A limited intensity of the signal was detected in 2.66 ± 1.15% of examined cells, and in 2.00 ± 1.00% of cells, the spot was absent. Conversely, in SH-SY5Y cells treated with DTX (1.25 nM), an intense localisation of tubulin was only in 10.33 ± 2.51% of cells, while a limited fluorescence was in 76.03 ± 1.01% of cells (Figure 2(b)), and the filamentous structure throughout the cytoplasm appeared lost in 10.33 ± 2.51% of analysed cells. Data obtained in untreated cells were significantly different from those obtained in the treated SH-SY5Y cells (absent fluorescent signal *P* = 0.002; limited fluorescent signal *P* < 0.001; intense fluorescent signal *P* < 0.001).

**Figure 3.**
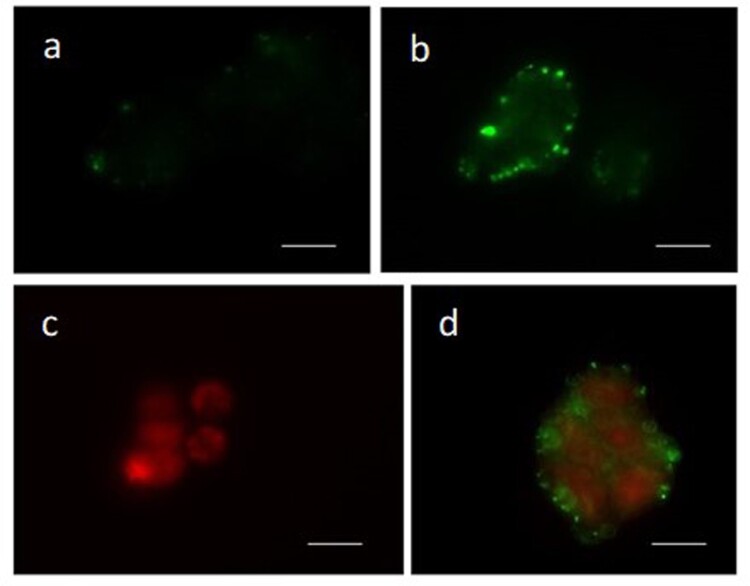
Annexin-V/Propidium Iodide assay in the neuroblastoma SH-SY5Y cells. Panel a) untreated cells; panels b–d) cells treated with DTX. Apoptotic (green, panel b), necrotic (red, panel c), and apoptotic/necrotic cells (green/red, panel d) are shown by Annexin-V/Propidium Iodide assay. Bar 20 μm.

The Annexin-V and Propidium iodide (AnV/PI) assay showed the apoptotic green signal and/or the necrotic red signal in only 5% of untreated cells (Figure 3(a)), while more than 60% of SH-SY5Y cells treated with DTX (Table 1, Figure 3(b–d)) highlighted these spots (Table 1). Early apoptotic cells were annexin V positive/PI negative, apoptotic/necrotic cells were annexin V positive/PI positive or annexin V negative/PI positive, and viable cells were annexin V negative/PI negative. Significant increased percentages of necrosis and apoptosis were detected in SH-SY5Y cells treated with DTX compared to those in untreated cells (Table 1).

**Table 1.** Quantitative analysis of apoptosis evaluated by Annexin-V and Propidium iodide assay in the SH-SY5Y cells.

	AnV−/PI− no fluorescent signal (%)	AnV+/PI− green fluorescent signal (%)	AnV−/PI+ red fluorescent signal (%)	Anv+/PI+ green/red fluorescent signal (%)
Untreated SH-SY5Y cells	95.33 ± 1.15	2.0 ± 1.0	0.66 ± 0.57	2.0 ± 1.0
SH-SY5Y cells treated with DTX 1.25 nM	26.66 ± 1.52	38.66 ± 0.57	8.66 ± 0.57	26.16 ± 1.20
One-way ANOVA test	*P* < 0.001	*P* < 0.001	*P* < 0.001	*P* < 0.001

Legend: AnV/PI, Annexin-V/Propidium iodide assay; AnV−/PI-, annexin-V negative/PI negative; AnV+/PI−, annexin-V positive/PI negative; AnV−/PI+ annexin V negative/PI positive; Anv+/PI+, annexin-V positive/PI positive. Green signal is index of early apoptosis; red and green/red fluorescent signal is index of apoptosis/necrosis, no fluorescent signal is index of viable cells. Data are expressed as mean values ± standard deviations. Statistically significant comparisons are in bold.

**Figure 4.**
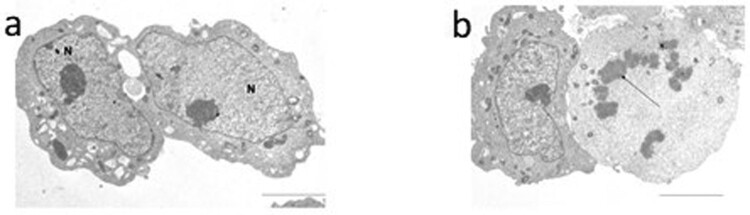
Transmission electron micrographs of the neuroblastoma SH-SY5Y cells. In panel a untreated cells are shown: the cells have regular nuclei (N) and organised cytoplasm. In panel b cells after DTX treatment are displayed: on the right an apoptotic cell is shown. Marginated chromatin is evident (arrow); the cytoplasm is devoid of organelles. Bar 12 μm.

According to TEM analysis, the untreated SH-SY5Y cells showed a normal ultrastructure, and the nuclei showed a regular chromatin condensation and well-organised cytoplasm (Figure 4(a)). In treated cells with DTX, TEM examination (Figure 4(b)) detected an increased percentage of cells (64 ± 2%; *P* < 0.001), showing apoptotic features such as marginated chromatin and vacuolisation compared to untreated ones (7.33 ± 1.52, Table 1).

### 3.3. Lipid oxidative damage

**Figure 5.**
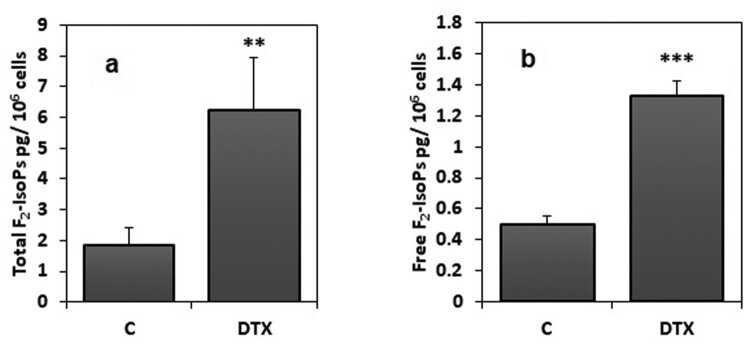
Total and free F_2_-IsoPs levels in DTX-treated and control cells. DTX treatment was 1.25 nM for 6 h. Data were means ± standard deviations of nine samples; **P < 0.01; ***P < 0.001 compared to control (C) samples (Student’s test). Legend: C, control (untreated) cells; DTX docetaxel, F_2_-IsoP, F_2_-isoprostanes

As an index of oxidative lipid damage, total F_2_-IsoPs (sum of free and esterified forms), were measured in cell samples as previously described. To evaluate fatty acid oxidative damage, 8-iso-prostaglandin F_2α_, one of the most represented isomers for F_2_-IsoP measurement, was detectable and measurable in all the analysed samples. In the SH-SY5Y cells, DTX treatment (1.25 nM for 6 h) induced a significant (*P* < 0.05) increase of both total F_2_-IsoPs (sum of unesterified molecules and esterified in phospholipids F_2_-IsoPs) and free F_2_-IsoPs (unesterified molecules only), as compared to control sample (SH-SY5Y cells incubated in the absence of DTX or with vehicle) (Figure 5; panels a and b, respectively). Moreover, statistical analysis showed a negative correlation between total F_2_-IsoPs levels and cellular viability (Spearman *r* = −0.754; *P* < 0.05). In addition, a negative correlation was observed between levels of total F_2_-IsoPs and ascorbic acid (Spearman *r* = −0.771; *P* < 0.05). Finally, a positive correlation between total F_2_-IsoPs levels and GSSG amounts (Spearman *r* = 0.943; *P* < 0.001) was detected.

### 3.4. Cellular antioxidant defense

**Figure 6.**
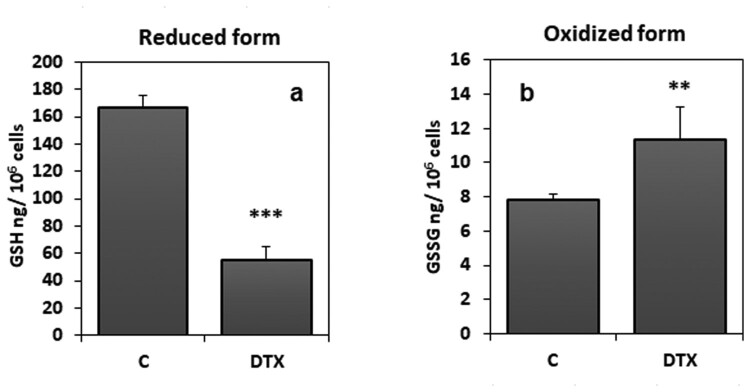
Reduced and oxidised glutathione levels in DTX-treated (1.25 nM, 6 h) and control cells. Data were means ± standard deviations of nine samples. **P < 0.01; ***P < 0.001 compared to control values (Student’s test). Legend: C, control (untreated) cells; DTX docetaxel, GSH, reduced glutathione; GSSG, oxidised glutathione.

**Figure 7.**
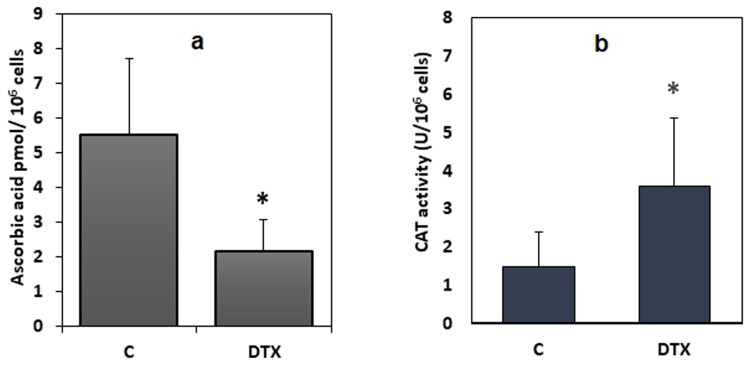
Levels of ascorbic acid, and catalase activity in DTX-treated (1.25 nM, 6 h) and control cells. Data were means ± standard deviations of nine samples. *P < 0.05 compared to control values (Student’s test). Legend: C, control (untreated) cells; DTX docetaxel.

Evaluation of enzymatic and non-enzymatic parameters of the cellular antioxidant defense system was showed (Figures 6 and 7). The levels of tripeptide glutathione in its reduced form, as shown in Figure 6(a), were statistically reduced in cells treated with DTX (1.25 nM, 6 h) by 38% (*P* < 0.001) compared to cellular levels of the control group. Moreover, a significant reduction in the GSH/GSSG ratio (*P* < 0.001), as an indication of redox imbalance, was observed. Additionally, statistical analysis showed a positive correlation between GSH/GSSG ratio and cellular viability (Spearman *r* = 0.665; *P* < 0.05).

Similarly, a statistically significant decrease (−39%) in the intracellular levels of ascorbic acid (*P* < 0.05) was shown (Figure 7(a)). Conversely, catalase activity (Figure 7(a)), a further enzymatic antioxidant defense system, was significantly increased in cells exposed to the drug, when compared to catalase activity in the control cells (*P* < 0.05).

## 4. Discussion

Since the role of long-term side effects of chemotherapy in causing cognitive impairment is widely debated, our intent was to verify whether DTX could damage brain cells, as non-targeted cells. Hence the choice of neuroblastoma cells on which the study was conducted.

In particular, the novelty of the study lies in demonstrating the occurrence of oxidative cellular damage caused by a relative low dose of DTX that could reach non-targeted cells throughout the duration of a chemotherapy treatment.

Free radical-mediated oxidative stress in the brain appears to be linked to the chemotherapy-induced cognitive impairment (CICI) affecting a significant fraction of cancer survivors [40]. The DTX-induced cognitive impairment is known and studied in animal model [41].

The antineoplastic activity of DTX acts by promoting the aggregation of tubulin into stable microtubules and inhibiting their disaggregation, thus leading to microtubule over-polymerisation, decrease in free tubulin, and ultimately to death by apoptosis [42]. Cytoskeleton is a dynamic structure in which the different components (microfilaments, microtubules, and intermediate filaments) are interconnected; within such a milieu, the action of DTX may be associated with the alteration of microtubular distribution, highlighted in our paper by immunofluorescence analysis. Unlike other chemotherapy drugs, the anticlastic activity of DTX does not affect mitochondrial functionality. Therefore, it is not easy to establish a relation between DTX and an increase in free radical species. Nevertheless, the anti-survival effect of DTX is associated with increased free radicals production [43].

Particularly perplexing is the fact that CICI is often associated with reactive oxygen species (ROS) generated by chemotherapeutic agents that do not cross the blood–brain barrier (BBB): so-called non-BBB-penetrating chemotherapeutic agents [8]. In this regard, members of the tubulin family were identified as potential contributors to the destabilisation of the BBB by DTX [44]. A possible interpretation of chemotherapy-related cognitive decline could take into consideration the capacity of the drug to cross the BBB. In this regard, *in vivo* passage of DTX through BBB is extremely difficult due to the physicochemical and pharmacological characteristics of the drug [45]. Nevertheless, in DTX passage could be involved the presence of transporters localised on the BBB. The genetic polymorphisms and the activity of ATP-binding cassette (ABC) transporters can be a mechanism that underlies the adverse response on CNS of anticancer therapies. A chronic therapy that causes the achievement of low-concentration docetaxel on CNS may cause an increase of side effects such as chemobrain. Moreover, the inhibition of P-glycoprotein (Pgp) caused by drug interactions could increase the accumulation of DTX in the brain [46]. Finally, the aging influence should be considered for the penetration and the passage of the BBB [47].

Nevertheless, although alterations of BBB properties upon DTX treatment *in vivo* studies were not reported, *in vitro* assays revealed a temporary DTX-related barrier disruption [44]. The occurrence of oxidative brain damage has also been investigated for other chemotherapy drugs that do not cross the BBB, involving inflammatory-mediated oxidative stress in the brain prevented by antioxidant scavengers [8]. In addition, researchers in this field need to take into account that repeated administration of DTX in chemotherapy protocols could promote the crossing of the BBB. According to the very low BBB permeability of DTX, a sublethal dose of DTX was tested in our study to better reflect the probable interaction *in vivo* between DTX and cells in the nervous tissue.

In this study, the neuroblastoma SH-SY5Y cells were used because they represent an accepted cell model of oxidative stress-induced neuronal cell death in chronic neurodegenerative diseases [48–50]. Although similar experiments could have been conducted in human cancer cell lines of glioblastoma (DBTRG-05MG), it was taken into account that in cerebral diseases the role of glia, as passive responders to neuronal damage rather than drivers of synaptic dysfunction, is still debated [51]. In addition, DTX dose and time of incubation were selected on literature information. In this regard, Riccardi et al. [52] calculate the IC50 of the DTX in SH-SY5Y cells, which turns out to be 10 nM after 48 h. In the basis of the viability data, the dose employed in our study was satisfactory for the purpose. The choice of short-term exposure to a 10 times lower dose was made to avoid the known cytotoxic effect of the drug.

In the investigated cellular system, the increased F_2_-IsoP amount in DTX-treated cells is attributable to the effect of ROS on membrane phospholipids. By considering that F_2_-IsoPs are generated by the free radical attack of esterified arachidonic acid, the measured total F_2_-IsoPs, as the sum of the free and esterified in phospholipids, are considered an index of membrane lipid peroxidation [53]. In the cellular system, the detection of total F_2_-IsoPs appears to be the best approach to evaluate isoprostane formation and the relevance of total isoprostanes to the evaluation of oxidative damage in the cellular system has been defined [54]. Moreover, in our cellular model the detected F_2_-IsoPs appear to be almost all esterified to phospholipids, since F_2_-IsoPs in free form represent only about 25% of total isoprostanes. On the same line, a meta-analysis showed that total F_2_-IsoP levels in plasma are about 10 times the amounts of free F_2_-IsoPs [55]. Since IsoP-containing phospholipids have proven to be structurally distorted molecules [56], alteration of membrane fluidity and integrity would be expected.

Brain cell susceptibility to oxidative damage has been linked to enzymatic activities able to counteract the accumulation of free radicals [57]. Accordingly, in our cellular model a modulation of the antioxidant response appears to be linked to DTX action, using a sublethal dose. In particular, the decrease in ascorbic acid levels is counterbalanced by an increase in catalase activity. Such a phenomenon could be discussed as a cellular adaptive response to hinder oxidative stress damage [58]. Catalase, one of the crucial antioxidant activity that mitigates oxidative stress, has a central role among the proteins involved in the fatty acid oxidation pathway, the amino acid metabolism pathway, and responses against oxidative stress [59]. Specifically, catalase activity has been included in the antioxidant up-regulation following a sublethal oxidant stimulus [58], and it has been reported that neuroectodermal cells present elevated basal activity of enzyme catalase [60]. In addition, enhanced catalase expression has been described as an adaptation to the hypoxia-induced lipid peroxidation [61]. Accordingly, catalase activity has been reported to be a biomarker for mild-stress-induced robustness [62]. Interestingly, an up-regulated expression of catalase has been reported in the SH-SY5Y cells as a neuroprotective effect of noni juice [63]. The relevance of our results for catalase activity is reinforced when it is considered that an increase in the activity of catalase occurs when cells undergo apoptosis [64,65]. Nevertheless, the mechanisms controlling the transcription of the catalase gene are poorly understood, and diverse mechanisms have also been proposed to regulate catalase expression [66]. Additionally, in some conditions, low catalase activity, as well as the decreased glutathione amount, contribute to oxidative stress [67]. Thus, our data of catalase activity appear to mirror a modulation of the antioxidant response induced by a cellular adaptation response to DTX-induced oxidative stress. Such modulation in antioxidant response (decreased ascorbic acid level and increased catalase activity) reinforce our choice of the DTX dose on account of its being able to induce mild oxidative stress injury without an extensive/unspecific cellular damage.

The decrease of ascorbic acid in the cells exposed to DXT could be due to the increased requirement of reducing equivalents needed to maintain GSH levels through the ‘sparing’ effect of AA on GSSG [68]. In SH-SY5Y cells treated with DXT the demand for reducing equivalents was not satisfied, possibly due to the excess of ROS and to the fact that GSH remained below the value detected in cells of the control group.

Apoptosis, a physiological form of cell death, and a pathway for regulating homeostasis and morphogenesis of cells, is associated with various diseases, particularly cancer. Certainly, apoptosis is widely recognised as an innate cell defense against carcinogens [69]. In our study, apoptosis was detected by ultrastructural analysis (cells with blebbing, shrinkage, altered chromatin condensation) and by Annexin V-Propidium iodide assay. Both tests confirmed a pro-apoptotic action of DTX in treated cells. DTX induces apoptosis in cancer cells, as demonstrated by associated changes in the mitochondrial membrane potential and overexpression of BCL2. Although the activation of caspase 8 and BID (tBid) seems to be a late event, apoptosis is induced by the activation of caspase 2, which initiates mitochondrial-dependent apoptosis through activation of Bax (direct or indirect) [70].

Tao et al. [71] reported that the administration of DTX on oral squamous cell carcinoma cell lines (KB cells) induces apoptosis through the mitochondrial pathway. The authors highlight an increase in the p53 protein that reduces intracellular GSH, which is also reduced by ROS due to its scavenger antioxidant function. This high decrease in GSH tripeptide induces the opening of mitochondrial permeability transition pores and a consequent decrease in mitochondrial membrane potential. Finally, the Bax protein is transferred to the mitochondrion and causes the release of cytochrome C from the mitochondrion to the cytoplasm, thus activating the caspase signal and, apoptosis of the KB cells occurs. Recently, Singh et al. [72] showed that DTX combined with thymoquinone induces apoptosis in prostate cancer cells via inhibition of the PI3 K/AKT signaling pathway, improving the survival rate and quality of life of prostate cancer patients.

The evidence obtained from the investigations of apoptosis and oxidative stress appear to be especially relevant to that mechanism involved in cognitive impairment, and are recognised as relevant factors in CICI. Interestingly, inhibition of apoptosis and oxidative stress has been shown to be relevant in improving neuroprotection against doxorubicin-induced chemobrain [73]. Additionally, tubulin, which here has been demonstrated to be involved in DTX-induced neuron damage, is a key element in modulating hippocampus efficacy in cisplatin-treated mice [74].

## 5. Conclusions

Our findings indicate that exposure of the SH-SY5Y cells to a DTX sublethal dose that can mimic the condition of non-targeted brain cells in chemotherapy triggers a redox imbalance linked to cytoskeletal changes and lipid peroxidation. This study provides a global evaluation of the condition of oxidative stress. Such new evidence is a useful contribution to the knowledge applicable in the evaluation of antioxidant treatment during chemotherapy, which has been evaluated, in clinical trials as well, with the intention of reducing toxic side effects.

## Abbreviations

**Abbreviations:** AnV: annexin-V; AnV/PI: annexin V/propidium Iodide; BBB: blood brain barrier; CAT: catalase; CICI: chemotherapy induced cognitive impairment; DTX: docetaxel; F_2_-IsoPs: F_2_-isoprostanes; GSH: reduced glutathione; GSSG: oxidised glutathione; MTT: cell-proliferation assay; 3-(4,5-dimethylthiazol-2-yl)-2,5-diphenyltetrazolium bromide compound; PI: propidium iodide; TEM: transmission electron microscopy
